# Characterization of the complete chloroplast genome of *Oxalis corymbosa* DC. (Oxalidaceae), a medicinal plant from Zhejiang Province

**DOI:** 10.1080/23802359.2021.1882905

**Published:** 2021-03-22

**Authors:** Manjia Zhou, Meixiu Yan, Zhiqi Ying, Xiangyu He, Yuqing Ge, Rubin Cheng

**Affiliations:** a College of Pharmaceutical Science, Zhejiang Chinese Medical University, Hangzhou, China; b The First Affiliated Hospital of Zhejiang, Chinese Medical University, Hangzhou, China

**Keywords:** *Oxalis corymbosa*, complete chloroplast genome, phylogenetic analysis, simple sequence repeats

## Abstract

*Oxalis corymbosa* DC. is an important medicinal and edible perennial herb belonging to the wood-sorrel family Oxalidaceae. In this study, we report the complete chloroplast (cp) genome sequence of *O. corymbosa*. The assembled chloroplast genome was 151,351 bp in length, containing two inverted repeated (IR) regions of 24,587 bp each, a large single copy (LSC) region of 85,476 bp, and a small single copy (SSC) region of 16,701 bp. The genome encodes 128 genes, consisting of 82 protein-coding genes, 37 tRNA genes, eight rRNA genes, and one pseudogene (*ycf1*). The 82 protein-coding genes encode 25,751 amino acids in total, most of which use the initiation codon ATG, except *rps19* and *psbC* genes start with GTG. The lengths of the tRNA genes range from 71 bp to 93 bp, with the highest GC content of 62.16% in tRNA-Arg (ACG). The overall GC content of *O. corymbosa* is 36.47%, with the highest GC content of 42.64% in IR region. In addition, a total of 74 simple sequence repeats were identified in the cp genome of *O. corymbosa*. Phylogenetic analysis indicated a sister relationship between *O. corymbosa* and *O. drummondii*, suggesting a close genetic relationship between the two *Oxalis* species. This work provides basic genetic resources for investigating the evolutionary status and population genetics diversities for this medicinal species.

*Oxalis corymbosa* DC. is an important medicinal and edible perennial herb and widely distributed throughout the world. This species was introduced into China as an ornamental plant in the mid-19th century and is now abundantly found in agricultural farms, gardens, and lawns (Tsai et al. 2020). *Oxalis corymbosa* was recorded as Tongchuicao in a variety of Local Records of Chinese Traditional Medicine with effects of removing blood stasis and detumescence, clearing heat, and removing dampness (Gao et al. 2011). The extracts of *O. corymbosa* have been demonstrated as a valuable natural source of antioxidants, suggesting potential applications in both medicinal and food industries (Liao et al. 2019). The antioxidant capacity of hydrophilic extracts from *O. corymbosa* was positively related with the total phenolic contents (Tukun et al. 2014). In addition, *Oxalis* is the largest genus of family Oxalidaceae, comprising of more than 500 species (Vaio et al. 2013). The taxonomy has been revised with similarities in phenotypes across different species (Lubna et al. 2020). The chloroplast genome (cp) has been proven to be a valuable resource for species identification and plant phylogenetic analysis (Wang et al. 2020). Furthermore, it is necessary to develop effective molecular identification strategy for *O. corymbosa* to ensure the safety of clinical application. The aim of this study is to analyze chloroplast genome sequence of *O. corymbosa*, which could contribute to the development of molecular markers and phylogenetic relationship investigation.

The sample of *Oxalis corymbosa* was collected from Fuyang area of Zhejiang Province (30°05′2.4″N, 119°53′20.4″E). The leaf specimen was deposited at Medicinal Herbarium Center of Zhejiang Chinese Medical University, Hangzhou, China (Voucher Identifying Number CPC-02). Total genomic DNA was extracted and sequenced using the Illumina Hiseq Platform according to the previous report (Dong et al. 2020; Gao et al. 2020). The chloroplast genome of *O. corymbosa* was assembled by metaSPAdes with the chloroplast genome sequence of *Oxalis drummondii* as reference (Nurk et al. 2017). Chloroplast genome sequence was annotated using GeSqe and further confirmed by BLAST (Tillich et al. 2017). The complete cp genome of *O. corymbosa* was submitted to GenBank with the accession number of MW057776.

The length of the complete chloroplast genome sequence of *O. corymbosa* was 151,351 bp, with a large single copy (LSC) region of 85,476 bp, a small single copy (SSC) region of 16,701 bp, and two separated inverted repeated (IR) regions of 24,587 bp each. A total of 128 genes were identified in the cp of *O. corymbosa*, including 82 protein-coding genes, 37 tRNA genes, eight rRNA genes, and one pseudogene (*ycf1*). The overall GC content was 36.47%, and the corresponding contents for LSC, SSC, and IR regions were 34.19%, 29.97%, and 42.64%, respectively. The genome included 15 duplicated genes in the IR region, including seven tRNAs, four rRNAs and four protein-coding genes. The proportion of coding sequences with a total length of 77,499 bp is 51.2%, which encodes 25,751 amino acids. The most frequently used amino acids were Leu (10.6%), followed by Ile (8.8%), Ser (7.7%), Gly (6.6%), and Phe (5.9%), respectively. Most of the protein-coding genes in *O. corymbosa* started with a typical ATG codon, except for *rps19* and *psbC* genes that used the initiation codon GTG. For the stop codon, 44 of 82 genes ended with TAA, 20 protein-coding genes ended with TAG, while the other 18 genes terminated with TGA. It is interestingly to note that one of *ycf1* is a pseudogene that has only partial fragment. Similar to most chloroplast genomes, the space gap of nucleotides are very common in *O. corymbosa*. The tRNA genes of *O. corymbosa* vary from 71 to 93 nucleotides, with the GC content range from 39.73% to 62.16%. Moreover, a total of 74 small single repeats (SSR) are identified in the cp of *O. corymbosa*, ranging from 10 bp to 131 bp.

The ML tree was inferred by MEGA 7.0 using the newly determined complete genome sequence of *O. corymbosa* as well as the cp sequences of other eight representative species from the family Oxalidaceae. The result demonstrated that *O. corymbosa* was clustered together with *O. drummondii*, suggesting a close genetic relationship between the two species (Figure 1). In addition, the three species from genus *Oxalis* formed a monophyletic group, and exhibited a sister relationship with *Averrhos carambola.* The four species combined together to form the group of Clade I (Figure 1). Our results would contribute to the development of molecular markers and further investigation on the population genetics and phylogenetics of the genus *Oxalis*.

**Figure 1.**
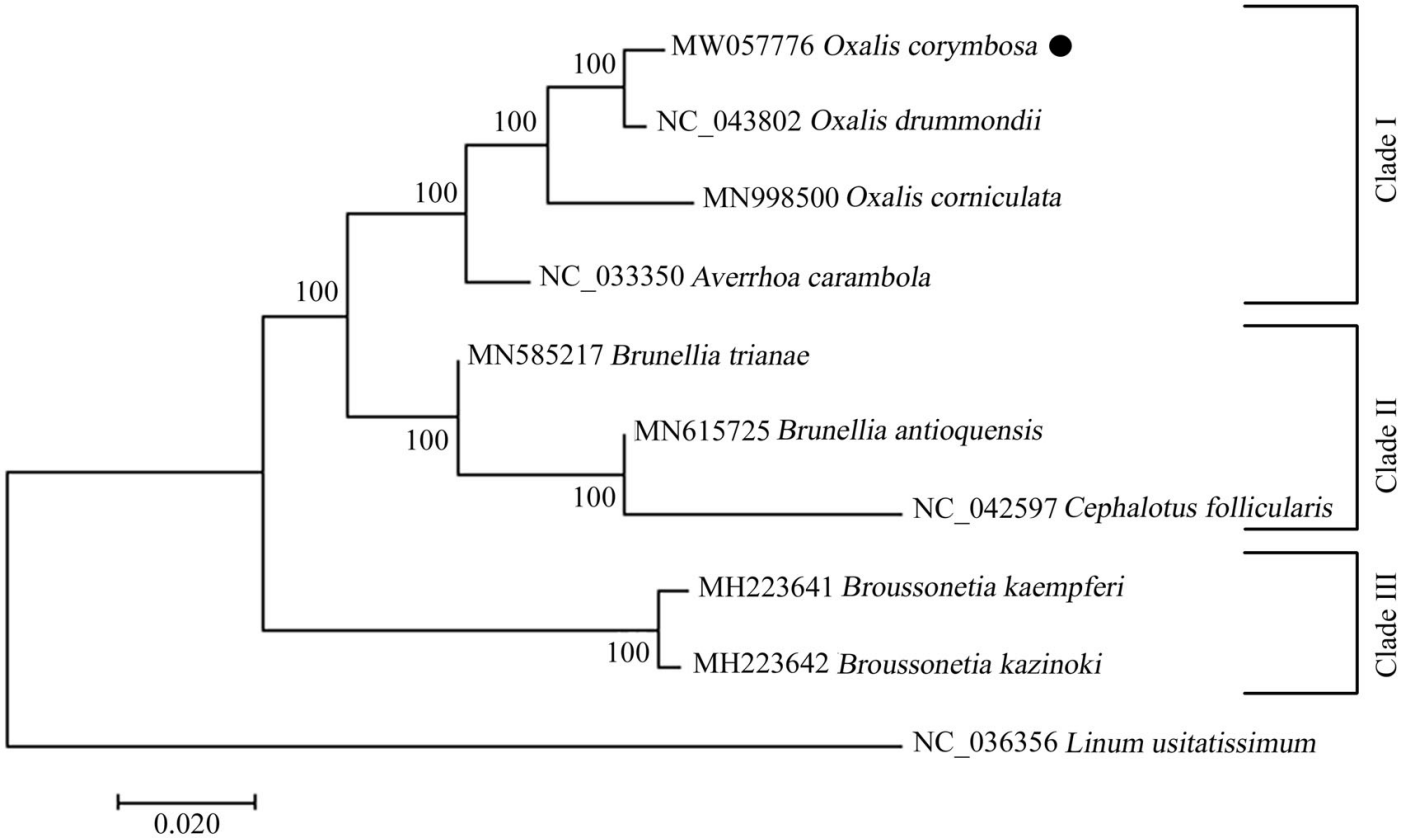
Phylogenetic relationships of the newly sequenced *Oxalis corymbosa* and other representative species from family Oxalidaceae based on the complete chloroplast genomes. The tree was generated by MEGA 7.0 with maximum-likelihood (ML) method. The species of *Linum usitatissimum* was chosen as the out-group. The newly determined genome of *O. corymbosa* was indicated with black circle. Numbers on the nodes represent bootstrap values from ML analysis with 100 replicates. The GenBank accession numbers were listed before the species name.

## Data Availability

The genome sequence data that support the findings of this study are openly available in GenBank of NCBI at (https://www.ncbi.nlm.nih.gov/) under the accession no. MW057776. The associated BioProject, SRA, and BioSample numbers are PRJNA690071, SRR13370090 and SAMN17227902, respectively.

## References

[CIT0001] Dong S, Gao C, Wang Q, Ge Y, Cheng R. 2020. Characterization of the complete chloroplast genome of *Macleaya cordata* and its phylogenomic position within the subfamily papaveroideae. Mitochondrial DNA Part B. 5(2):1714–1715.

[CIT0002] Gao C, Wang Q, Ying Z, Ge Y, Cheng R. 2020. Molecular structure and phylogenetic analysis of complete chloroplast genomes of medicinal species *Paeonia lactiflora* from Zhejiang Province. Mitochondrial DNA Part B. 5(1):1077–1078. 33366882 10.1080/23802359.2020.1721372 PMC7748428

[CIT0003] Gao PL, Guo SL, Lou YX. 2011. Adaptation characteristics of *Oxalis corymbosa* and *O. triangularis* to light factor based on Chlorophyll fluorescence parameters. J Shanghai Normal Univ (Nat Sci). 40(5):528–532.

[CIT0004] Liao XY, Xu HM, Feng P, Wang YX, Huang JY. 2019. Evaluation of *Oxalis Corymbosa* extracts from different plant parts and seasons as a potential source of antioxidants. Curr Top Nutraceutical Res. 17:50–55.

[CIT0005] Lubna Asaf S., Jan R, Khan AL, Lee IJ. 2020. Complete chloroplast genome characterization of *Oxalis Corniculata* and its comparison with related species from family Oxalidaceae. Plants. 9:928. 10.3390/plants9080928 PMC7464629 32717796

[CIT0006] Nurk S, Meleshko D, Korobeynikov A, Pevzner PA. 2017. metaSPAdes: a new versatile metagenomic assembler. Genome Res. 27(5):824–834. 28298430 10.1101/gr.213959.116 PMC5411777

[CIT0007] Tillich M, Lehwark P, Pellizzer T, Ulbricht-Jones ES, Fischer A, Bock R, Greiner S. 2017. GeSeq – versatile and accurate annotation of organelle genomes. Nucleic Acids Res. 45(W1):W6–W11. 28486635 10.1093/nar/gkx391 PMC5570176

[CIT0008] Tsai MY, Jane WN, Chen SH, Kao WY. 2020. Female sterility and ovule malformation of *Oxalis corymbosa*, an exotic plant in Taiwan. Flora. 265:151571.

[CIT0009] Tukun AB, Shaheen N, Banu CP, Mohiduzzaman M, Islam S, Begum M. 2014. Antioxidant capacity and total phenolic contents in hydrophilic extracts of selected Bangladeshi medicinal plants. Asian Pac J Trop Med. 7:S568–S573. 10.1016/S1995-7645(14)60291-1 25312185

[CIT0010] Wang Q, Yu S, Gao C, Ge Y, Cheng R. 2020. The complete chloroplast genome sequence and phylogenetic analysis of the medicinal plant *Rubus chingii* Hu. Mitochondrial DNA Part B. 5(2):1307–1308. 10.1080/23802359.2019.1688115 PMC7707760 33366231

[CIT0011] Vaio M, Gardner A, Emshwiller E, Guerra M. 2013. Molecular phylogeny and chromosome evolution among the creeping herbaceous *Oxalis* species of sections Corniculatae and Ripariae (Oxalidaceae). Mol Phylogenet Evol. 68(2):199–211. 23562801 10.1016/j.ympev.2013.03.019

